# Effects of Acupressure on Fatigue and Depression in Hepatocellular Carcinoma Patients Treated with Transcatheter Arterial Chemoembolization: A Quasi-Experimental Study

**DOI:** 10.1155/2015/496485

**Published:** 2015-02-23

**Authors:** Su-Chen Lan, Yueh-E Lin, Shu-Ching Chen, Yu-Fang Lin, Yu-Jen Wang

**Affiliations:** ^1^Department of Nursing, Chang Gung Medical Foundation, Chang Gung Memorial Hospital, Linkou District 33305, Taiwan; ^2^Department of Nursing, Chang Gung University of Science and Technology, Taoyuan City 33303, Taiwan; ^3^School of Nursing, National Taipei University of Nursing and Health Sciences, Taipei City 11219, Taiwan; ^4^Administration Center of Nursing Department, Chang Gung Memorial Hospital, Taoyuan City 33378, Taiwan

## Abstract

This study was to examine the effects of acupressure on fatigue and depression in HCC patients undergoing TACE. A quasiexperimental study design was used. Patients were evaluated at five time points: before treatment (T1) and 2, 3, 4, and 5 days after treating TACE (T2, T3, T4, and T5). Fatigue and depression were assessed by a VAS fatigue scale and a VAS depression scale at each time point. TFRS and BDI were administered at T1 and T5. Patients' fatigue and depression were significantly higher at T5 than at T1 in two groups. Fatigue and depression increased in both the experimental and control groups' patients over the five days of hospitalization during which TACE and chemotherapy were administered. The experimental group had significantly less fatigue than the control group, with lower subscale scores on physical, psychosocial, daily, and overall fatigue. There were no differences between the groups on depression. At posttest, the experimental group experienced lower physical, psychosocial, daily, and overall fatigue than the control group. Acupressure can improve fatigue in HCC patients during treatment with TACE but did not alleviate depression. Discharge planning should include home care for management of fatigue and depression.

## 1. Introduction

Hepatocellular carcinoma (HCC) is the major cause of health problems in many developed countries and varies according to geographic location [1]. In Taiwan, HCC is the second most common cause of cancer death [2]. Transcatheter arterial chemoembolization (TACE) can be palliative for unresectable HCC [3]. Fatigue and depression are reported as the most common problems for HCC patients treated with TACE [4–7]. Unrelieved fatigue and depression may cause patients to withdraw from treatment and can negatively impact quality of life.

Some reports have shown that HCC patients have significant fatigue and depression gradually increasing during treatment. Lai et al. [8] noted that, in HCC patients, fatigue intensity peaked at 5 weeks of radiation therapy. Depressive status predicted fatigue intensity and sleep interference. Research also verified that Taiwanese patients with HCC receiving TACE experienced moderate levels of fatigue which peaked at 2 days after initial TACE [9]. In several studies [4–6, 10] of HCC patients receiving TACE, it was reported that fatigue was the most common adverse effect causing sleep disturbances [5], reduced physical and emotional status [11], and produced a negative effect on overall quality of life [12].

Alcohol consumption has been identified as being associated with hepatocellular carcinoma (HCC) [13]. Alcohol use may be a risk factor for developing depression [14, 15]. Depression is also a common distress factor in HCC patients undergoing TACE. Depression refers to characteristics of sadness, loss of interest, feeling of guilt or low worth, disturbed sleep or appetite, feelings of tiredness, and poor concentration which may occur during several weeks of stressful situation up to a period of several months. Depression has a substantial effect on work and daily lives, sleeping, and general health [16, 17]. Studies in HCC patients treated with TACE revealed that patients experienced variant depressive episodes during posttreatment [5, 7, 18, 19] and there was a strong correlation with fatigue [18] and sleep disturbance [5, 18], impacting quality of life [19].

Acupressure is a form of touch therapy that utilizes pressure with thumbs or fingertips to stimulate discrete points on the body for relief of a variety of symptoms and to mitigate tension or pain [20]. Acupressure is a practical intervention and complementary therapy for cancer-related fatigue and has also been highlighted in a critical research review [21]. In a previous study that applied acupressure to breast cancer survivors after completion of cancer treatments, the severity of fatigue and depression improved [22]. The massage-acupressure intervention also increased serenity and decreased depression for children with hematopoietic cell transplant [23]. A study on interventions using acupressure and transcutaneous electrical acupoint stimulation (TEAS) in patients receiving hemodialysis demonstrated that patients had significantly lower levels of fatigue and less depressed moods compared to patients in the control group [24]. Research supports the effectiveness of acupressure in alleviation of cancer-related fatigue [25, 26] and depressed mood [22–24]. Acupressure is a low cost, safe, and convenient traditional Chinese medicine (TCM) technique and a self-care intervention. The acupressure rationale was derived from TCM [27]. Key concepts in Chinese medicine state that the body is an energetic system in dynamic balance, containing yin (tendons, bones, and the internal organs), yang (skin and the external portion of the body), and qi (energy and meridians). Acupressure, by stimulating specific points, promotes the circulation of qi, opens up the meridian channels influencing energy and mood, and attempts to bypass blockage of vital flow collateral meridians at specific points to restore the balance of yin and yang [28, 29]. There are few studies involving acupressure to provide improvement in fatigue and depression in HCC patients treated with TACE. The aim of this study was to explore the effects of acupressure on fatigue and depression in HCC patients treated with transcatheter arterial chemoembolization.

## 2. Materials and Methods

### 2.1. Study Population and Design

This study was a quasi-experimental study with nonrandomized two groups, using a pre- and posttest design. Sixty-two participants were recruited from gastroenterology inpatient wards in a medical centre in northern Taiwan. Participants met the following inclusion criteria: (1) having HCC as diagnosed by the gastroenterologist, (2) age ≥20 years, (3) being treated with TACE, and (4) willingness to participate in this study. Exclusion criteria were as follows: (1) any unstable systemic disease (heart disease, hypertension, hypotension, and active bleeding), (2) skin defect or infection, (3) any other condition likely to cause discomfort during TACE (fever, pain, nausea, vomiting, etc.), and (4) being with prior treatment (cumulative treatment effects could influence outcomes).

### 2.2. Procedures

**Figure 1:**
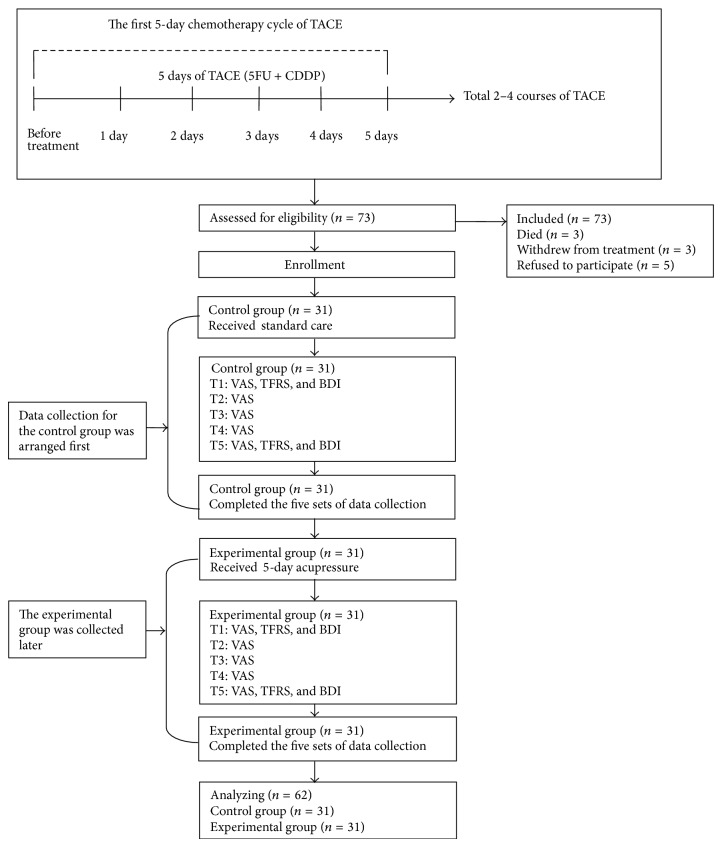
The flowchart of recruiting and TACE protocol.

Before the study was conducted, it was approved by the Institutional Review Board of the study hospital. Written informed consent was obtained from the participants before collecting data. Subjects were randomized to groups because all patients at Chang Gung Memorial Hospital were treated with the same TACE protocol. To prevent contamination the nonrandomized assignment was used in this study, data collection for the control group was arranged first, and the experimental group was collected later. The control group received standard care, and the experimental group received acupressure (Figure 1).

Data were collected at five time points: before treatment (T1) and 2, 3, 4, and 5 days after receiving the first course of chemotherapy as part of the transcatheter arterial chemoembolization (TACE) process (T2, T3, T4, and T5). Patients' personal and clinical characteristics were collected at baseline (T1). Patients' fatigue and depression were assessed by visual analogue scales at each time point. Considering participants' interview burdens, the assessment instruments (Tang Fatigue Rating Scale [30] and Beck Depression Inventory [31]) were measured at T1 and T5.

The TACE schedule was 2–4 courses of transcatheter hepatic arterial embolization based on treatment guidelines for HCC. A fluorouracil (5FU) and cisplatin-based regimen was administered as an intravenous infusion daily, 5 days per week, for 2–5 weeks [32]. Patients in this study were in their first 5-day chemotherapy cycle of TACE, which included intravenous 5FU and cisplatin (CDDP) followed by TACE; patients remained hospitalized throughout the five days (Figure 1).

### 2.3. Interventions

**Figure 2:**
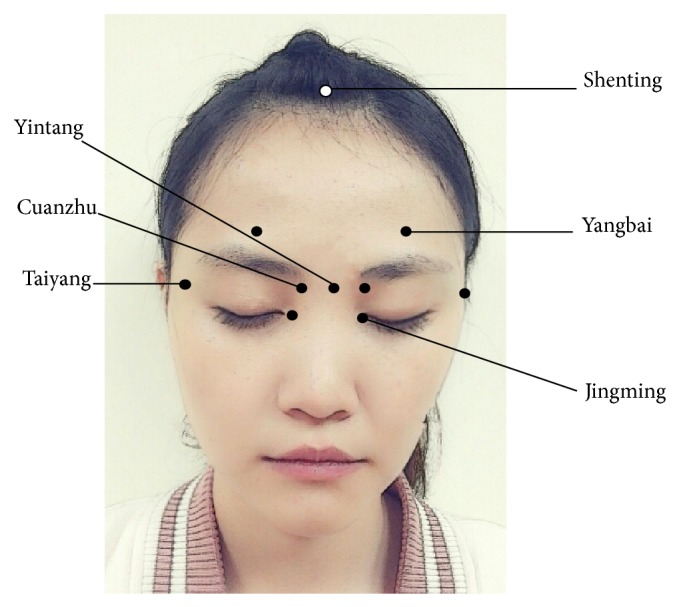
Acupoints (Yintang, Shenting, Cuanzhu, Taiyang, Jingming, and Yangbai) selected for fatigue and depression in the study.

**Figure 3:**
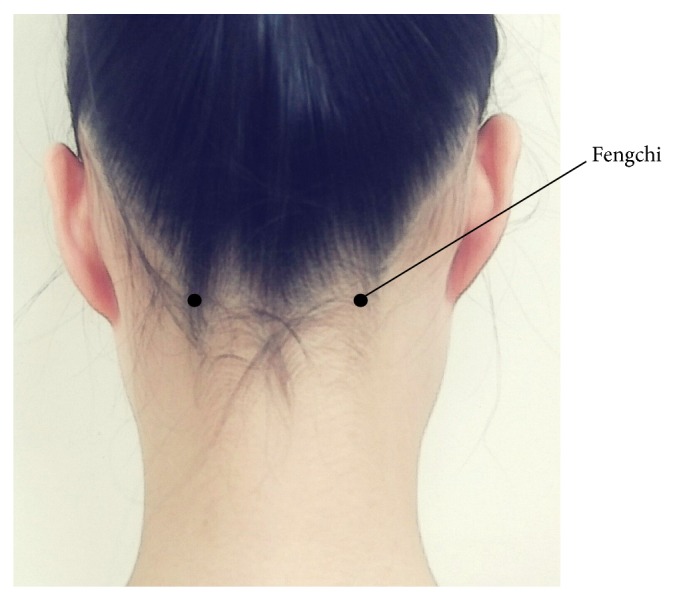
Acupoint (Fengchi) selected for fatigue and depression in the study.

**Figure 4:**
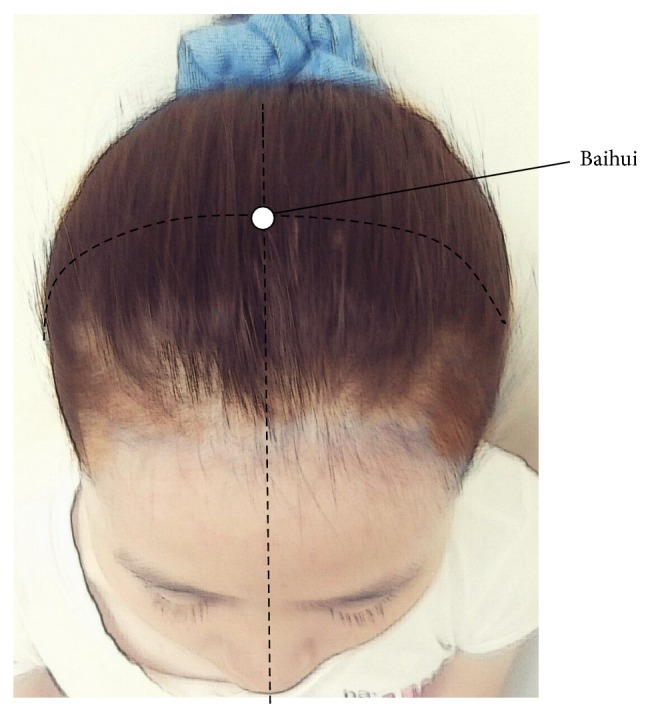
Acupoint (Baihui) selected for fatigue and depression in the study.

Acupressure contains eight auricular acupoints, including Yintang, Shenting, Cuanzhu, Taiyang, Jingming, Yangbai (Figure 2), Fengchi (Figure 3), and Baihui (Figure 4) [28]. Stimulating the Yintang, Cuanzhu, Taiyang, Yangbai, Fengchi, and Baihui acupoints can alleviate headache symptoms [33–35]. Stimulation of the Shenting acupoints can raise vitality and improve dizziness [33–35]. Stimulation of the Jingming acupoint can diminish tired eyes [33–35]. Stimulation of the Fengchi acupoint can improve stiff neck, headache, dizziness, and fatigue [33–35].

Participants in the control group were recruited from patients who received TACE. The control group received standard care during treatment. After completing data collection in the control group, participants in the experimental group were recruited. The acupressure program was provided to the experimental group.

Patients in the experimental group received a total of 5 days of acupressure, administered 2 times per day over a week's time frame. The duration of each acupoint massage was limited to 4 minutes. The researchers were trained for acupressure by a traditional Chinese physician from the Department of Acupuncture and TCM of the study hospital with expertise in the treatment of complementary and alternative therapies for cancer patients who has practiced acupuncture independently for more than 10 years. The research nurse had 18 hours of lessons and 36 hours of practical training in TCM. The training included information about the concept of acupressure, the sites of auricular acupoints, and the skills of acupressure. After instructions and practice, the research nurse completed a return demonstration to verify skills. An Approved TCM certificate was issued to the research nurse indicating knowledge of the discipline and practical application. To facilitate consistency, the traditional Chinese physician and the research nurse administered the pre- and posttests and interventions to the same groups of subjects in the pilot study. Before the start of the intervention, the set of questionnaires was administered to all participants the day before treatment (T1). Daily fatigue and depression were assessed at T2 to T5 every afternoon 10 minutes following daily acupressure. At T5, the questionnaires were administered to the two groups at the same time.

### 2.4. Measures

#### 2.4.1. Tang Fatigue Rating Scale (TFRS)

The TFRS, developed based on the critical dimensions and attributes of fatigue, was administered to measure fatigue [30]. The TFRS consists of 37 items on three subscales: physical, psychosocial, and daily activity. The items are arranged as a 1–10 scale, with higher scores representing a higher level of fatigue. The scale has shown good internal consistency and convergent validity in previous cancer studies [30, 36]. The Cronbach alpha for the TFRS in this study was 0.92.

#### 2.4.2. Beck Depression Inventory (BDI)

The BDI consists of 21 items with a 4-point response scale ranging from not at all to severe. Scores for the BDI range from 0 to 63. A higher score reflects more depressive symptoms [31]. The BDI has been widely used to measure depression in previous studies and is well-validated [30, 37]. Reliability of the scale was high with an internal consistency of >0.80.

#### 2.4.3. Visual Analogue Scale (VAS)

The VAS were developed to assess the intensity or severity of various symptoms [38]. The VAS is a line that is 100 mm long horizontally to measure a symptom, with the left side indicating “no such symptom at all” and the right side indicating “extreme severity of the symptom.” In this study, subjects were asked two questions relating to their subjective experience of fatigue/depression and were then asked to rate their fatigue severity/depression severity. The scales are validated tools that have been used in several studies [39–41]. Using a pilot study with a research nurse and a nursing professional, we selected 5 patients for assessing fatigue severity and depression severity and the interrater reliability of VAS were 0.99 and 098, respectively, demonstrating acceptable psychometric properties of the VAS in this study. Because the VAS scale is a subjective measure by the patient requiring the researcher to calculate a value, the measurement of interrater reliability is important.

#### 2.4.4. Personal and Clinical Information Form

Personal information on age, gender, occupation, marital status, educational level, and religion was collected. Clinical characteristics included time since HCC was diagnosed, type of hepatitis carrier, albumin level, and hemoglobin value.

### 2.5. Statistical Analysis

SPSS version 21.0 (Chicago, IL, USA) was used to analyze data. Descriptive statistics were used to analyze personal and clinical characteristics. Paired *t*-tests were used to examine the differences of depression and fatigue within group analysis between pretest and posttest scores in the experimental group and the control group. The difference in pretest and posttest values in fatigue and depression between group analyses was also examined by *t*-test. Repeated-measures analysis of variance (ANOVA) with post hoc analysis was employed to determine the differences in fatigue and depression at five different time points. A power analysis based on Cohen's [42] method indicated that 28 participants in each group were the minimum required for a two-group test of mean differences to achieve a power of 0.80, an effect size of 0.08, using an alpha of 0.05.

## 3. Results

Seventy-three patients met the inclusion criteria and were enrolled after written consent forms were obtained. Three patients died, three patients withdrew from treatment, and five patients refused the entire program because of physical discomfort. Therefore, a total of 62 patients participated in the study, with 31 in the control group and 31 in the experimental group.

### 3.1. Personal and Clinical Characteristics between Experimental Group and Control Group

The experimental group did not differ significantly from the control group in terms of age, gender, occupation, marital status, educational level, religion, time since HCC was diagnosed, type of hepatitis carrier, and albumin or hemoglobin levels. Within each group, the majority of participants were between 40 and 64 years of age, male, unemployed, married, with an elementary education level, held Buddhism or Taoism religious beliefs, and had been diagnosed within 1 year. The majority of the subjects (61.3%) were hepatitis B carriers. The average (SD) albumin was 3.34 (0.49) g/dL for the experimental group and 3.49 (0.57) g/dL for the control group. Hemoglobin was 11.47 (SD = 2.08) and 11.94 (SD = 1.94) for the experimental group and control group, respectively, without statistically significant differences (Table 1).

### 3.2. Differences of Fatigue and Depression by Group

The results of paired *t*-tests for differences in fatigue and depression between pretest and posttest in the subscale scores, the experimental group and the control group, respectively, are shown in Table 2. Posttest scores were all higher than pretest scores for the subscale scores.

### 3.3. Differences in Pretest and Posttest in Fatigue and Depression between Experimental Group and Control Group

For better understanding of the intervention causal effects on program participants in pre- (T1) and postintervention (T5), independent* t*-tests were utilized to examine differences in fatigue and depression between the two groups, which were measured by TFRS and BDI, respectively. Overall differences in pre- and postintervention in the control group were significantly higher than in the experimental group, especially for physical fatigue (*t* = 6.13, *P* < .001), psychosocial fatigue (*t* = 6.87, *P* < .001), daily fatigue (*t* = 6.64, *P* < .001), and overall fatigue (*t* = −6.13, *P* < .001). The control group had greater increase in fatigue over time than the experimental group. There were no differences between groups on depression (Table 3).

### 3.4. Change in VAS Fatigue and VAS Depression within Groups over 5 Times

Daily fatigue and depression levels, as measured by one-item VAS scores, increased for both groups over time. The fatigue experimental group was lowest at T1, increased from T2 to T5, and peaked at T5 (5 days from treating TACE initiation), with statistically significant differences over time. The change of fatigue in control group had a similar slope to the experimental group. The mean score for depression in the experimental group was lowest levels at T1 and T2, slightly increasing from T2 to T3, slightly dropping at T4, and peaking at T5 (5 days after TACE treatment initiation), with the difference being statistically significant. Control group patients' depression followed a similar pattern (Table 4).

## 4. Discussion

This study addressed the effects of acupressure on fatigue and depression in HCC patients treated with TACE. Several important findings were recognized. The changes in fatigue increased through the first five days of treating TACE over time, and physical fatigue was the worst level of fatigue across the three dimensions. Tang et al. [30] surveyed congestive heart failure patients and found daily activity fatigue caused the most distress during hospitalization. Results of the current study are inconsistent with those of previous studies, where decreasing daily activity implied insufficient heart function, which led to systemic limitations with poor physical function status. Fatigue has also been correlated with poor cardiac function, including decreased cardiac output, stroke volume, or ejection fraction, and narrowed or hardened arteries restricting blood flow to the muscles due to physical intolerance [43]. Subjects in our study may experience fatigue due to side effects of TACE and its chemotherapy. Advocacy to ensure adequate management of fatigue has become an essential component in clinical cancer care. Patients with HCC received 5 days of intravenous 5FU and CDDP followed by TACE without activity restrictions, including no restrictions on self-care and with encouraged walking during treatment. The improvement of fatigue, walking exercise, and strength training should be considered in patients with HCC treated with TACE.

In the present study, the patients participated in the acupressure program involving the eight auricular acupoints for 5 days during intravenous 5FU and CDDP followed by TACE. The researcher provided the acupressure intervention. The short- or long-term effects of acupressure have been identified to explain the length of consequences in other studies and the results were congruous [22–26]. Studies pointed to early effects of acupressure to improve fatigue and depression, but none was continued into later chemotherapy courses. Similar conclusions were shared by Zick et al. [22], who found 6-week self-administered acupressure led to significant improvements in depression, sleep quality, and fatigue among breast cancer survivors with persistent cancer-related fatigue. Mehling et al. [23] tested an acupressure-massage intervention and found that children with hematopoietic cell transplant who received the intervention reported feeling less tired and demonstrated decreasing depression for the time period of 7 days before to 21 days after transplant. Tsay et al. [24] reported that in a 1-month acupressure and TEAS program for patients undergoing hemodialysis, patients had significantly lower levels of fatigue, a better sleep quality, and less depressed moods. Sabouhi et al. [25] showed that 4-week acupressure intervention significantly improved total mean score of fatigue and fatigue mean scores in the behavioral, emotional, sensory, and cognitive dimension for hemodialysis patients. Zick et al. [26] stated a 12-week relaxation acupressure and found that cancer survivors who received the intervention reported greater reduction in fatigue than those who received high-dose stimulatory acupressure (HIS) and low-dose stimulatory acupressure (LIS). This study had shorter effects when compared with previous studies. Perhaps family caregivers could be trained to provide daily acupressure at home during the later courses of treatment to extend effects longer term.

The current study showed that patients with HCC who received acupressure for eight auricular acupoints improved fatigue and depression. This finding is inconsistent with a study by Tsay et al. [24] in which auricular acupoints were focused on the lower limbs. The fatigue characteristics reported by our participants were individual perceptions associated with distress and exhaustion, which were often temporary (duration of less than six months). In this study, auricular acupoints highlighted the head and neck area, so future studies should compare and test different specific pressure points in acute and chronic fatigue.

This study assessed daily fatigue and depression by VAS and pre- and posttests using self-reported questionnaires. Visual analogue scales are simple to administer and complete, highly sensitive, and reliable in rating various subjective experiences [43, 44]. Fatigue and depression are complex and multidimensional. The potential advantage is that the visual analogue scales only reflect intensity and cannot accurately describe responses in terms of patterns or characteristics [44, 45]. This allows patients to score their experience without external influence from preconceived values. The visual analogue scale is unidimensional, which could result in intervention effects not having sufficient significance to identify changes in daily fatigue and depression and making it difficult for healthcare providers to understand distressing phenomenon. Future studies need to consider different types of assessments (e.g., verbal analogue scales and semistructured questionnaires) to determine subtle changes in fatigue and depression.

Although we found depression improved slightly from acupressure, we did not find statistically significant differences between the two groups. In both groups, the depression at posttest (5 days after treating TACE initiation) was higher than depression at pretest (before TACE). Depression is a subjective feeling and is complex and multidimensional. Clinical and psychological status should be carefully assessed to determine patients' needs for psychological well-being. Acupressure assists with other interventions, such as focusing on activities of interest, maintaining a positive outlook, and communicating and discussing feelings with family members [46–48]. Depression has been identified as correlating with disease and treatment characteristics for cancer patients [49–51]. Continuing assessment of treatment effects and illness progress are still strongly suggested.

Our study also found that patients experienced highest levels of fatigue and depression at T5 (5 days after treating TACE initiation) in both groups. The level of fatigue was moderate; this finding was similar to previous studies [4, 11, 12], in which most cancer patients suffered from fatigue during and after treatment. The time point of peak fatigue and depression was at treatment completion and preparation for hospital discharge, and these distressing problems may impact physical function and daily living. Discharge planning should include plans for home care management of fatigue and depression, such as energy conservation discussions (e.g., planning ahead and organizing work, balancing activities, pacing oneself, identifying effects of environment, and prioritizing activities) [52], getting enough rest and sleep, having available family support, and gradually increasing activity over time.

## 5. Limitations and Recommendations

Although efforts were made to design a comprehensive study, there were limitations. In this study there was no sham control group, and experimental subjects may have received extra attention and effort. Previous study has been well documented that no statistically significant difference was seen between true and sham acupuncture-point stimulation [53]. Further studies incorporating a sham control group intervention and expanding sample power might clarify whether positive effects result from a placebo effect in the experimental group. Fatigue and depression were measured by VAS each day over research time frame. The excessive intensive measurement may cause interview burden. However, the five measurement time points were measured by different instruments. This may lead to an impact on the comparison of data. The study results should be interpreted with cause. Since albumin and hemoglobin are impacted by fatigue [54, 55], this may increase the level of depression in cancer patients [55]. Cachexia may occur as a result of cancer and its treatment. Anemia and low albumin are common symptoms of cachexia. Cachexia associated with reduced muscle metabolic and decreased physical performance and cause fatigue for cancer patients. Previous studies have reported that fatigue and depression may coexist in patients with cancer [52]. At posttest, there were insufficient funds in the study to test hemoglobin and albumin so the effects of their values have not been examined between pretest and posttest. Additional studies are needed to examine the significances of laboratory values (e.g., albumin and hemoglobin), fatigue, and depression over time during acupressure. This study follows patients' fatigue and depression from before treatment to 5 days after treating with TACE initiation which may limit interpretation of study findings at later time points after treatment. Longitudinal or long-term follow-up studies are needed to identify efficacy of acupressure on fatigue and depression. Another potential limitation may relate to other symptoms, such as neurovascular symptoms or pain; however, increased burdens to fatigue and depression and these outcomes are unlikely to differ. Further studies are warranted to investigate other symptoms derived from TACE that predispose HCC patients to fatigue and depression. The fact that this study used a quasi-experimental study design with nonrandomized sampling may also be a study limitation, as it may result in internal and external validity concerns that may make results not generalizable. Additional studies with randomized controlled trials are required. Confounding factors such as albumin and hemoglobin levels, dietary habits, and social support may have influenced treatment and caused a change in fatigue and depression. In the future, improved study design could provide additional avenues to explore the mechanism of acupressure impact on fatigue and depression in this population.

## 6. Conclusion

The study explored the effects of acupressure on fatigue and depression during transcatheter arterial chemoembolization in HCC patients. Findings indicate that acupressure can improve fatigue in HCC patients during the TACE treatment period; no improvement in depression was found. Both the control group and experimental group patients' fatigue and depression increased from T1 to T5 and peaked when this was final measurement point (5 days from treating TACE initiation). Discharge planning involving interventions to manage fatigue and depression are recommended. The acupressure program can be taught to caregivers guided by a standardised educational manual or use of multimedia to offer patients and families the opportunity to continue the program after hospitalization.

## Figures and Tables

**Table 1:** Personal and clinical characteristics by group (*N* = 62).

Characteristics	Experimental group (*N* = 31)	Control group (*N* = 31)	*χ* ^2^/*t*	*P*
	*N* (%)/mean (SD)	*N* (%)/mean (SD)		
Age			0.08	.96
>65	12 (38.7)	11 (35.5)		
64–40	14 (45.2)	15 (48.4)		
<40	5 (16.1)	5 (16.1)		
Gender			0.26	.20
Male	20 (64.5)	24 (77.4)		
Female	11 (35.5)	7 (22.6)		
Occupation			0.61	
Unemployed	19 (61.3)	17 (54.8)		
Employed	12 (38.7)	14 (45.2)		
Marital status			0.55	.50
Unmarried	1 (3.2)	2 (6.4)		
Married	30 (96.8)	29 (93.5)		
Education level			1.40	.92
None	8 (25.8)	8 (25.8)		
Elementary	11 (35.5)	10 (32.3)		
Junior high	3 (9.6)	4 (12.9)		
Senior high	9 (29.0)	8 (25.8)		
College and above	0 (0)	1 (3.2)		
Religion			0.30	.86
None	7 (22.6)	8 (25.8)		
Buddhism/Taoism	24 (77.4)	24 (77.4)		
Christianity/Catholicism	0 (0)	0 (0)		
Time since HCC was diagnosed			1.58	.81
<3 months	12 (38.7)	11 (35.5)		
<1 year	15 (48.4)	15 (48.4)		
2-3 years	3 (9.6)	2 (6.4)		
4-5 years	0 (0)	1 (3.2)		
>5 years	1 (3.2)	2 (6.4)		
Type of hepatitis carrier			0.17	.99
None	4 (12.9)	3 (9.6)		
B type	18 (58.1)	19 (61.3)		
C type	6 (19.4)	6 (19.4)		
B + C type	2 (6.4)	2 (6.4)		
Other	1 (3.2)	1 (3.2)		
Albumin	3.34 (0.49)	3.49 (0.57)	1.11	.27
Hemoglobin	11.47 (2.08)	11.94 (1.94)	0.91	.37

**Table 2:** Differences of fatigue and depression by group (*N* = 62).

Variable	Experimental group (*N* = 31)						Control group (*N* = 31)					
	Pretest		Posttest		*t*	*P*	Pretest		Posttest		*t*	*P*
	Mean	SD	Mean	SD			Mean	SD	Mean	SD		
Fatigue (TFRS)	44.81	15.11	81.90	37.42	−5.20	.001	43.16	13.42	113.52	57.00	−6.13	.001
Physical	18.52	5.63	34.42	14.01	−3.91	.001	17.35	3.93	43.35	24.17	−6.13	.001
Psychosocial	13.32	3.16	26.71	18.31	−3.96	.001	12.74	2.08	37.77	19.88	−6.87	.001
Daily activity	12.97	9.02	20.77	8.50	−5.20	.001	13.06	9.08	32.39	16.39	−6.64	.001
Depression (BDI)	1.50	3.05	8.23	7.80	−4.26	.001	1.57	3.11	6.93	7.25	−3.60	.001

TFRS: Tang Fatigue Rating Scale; BDI: Beck Depression Inventory.

**Table 3:** Independent *t*-tests analysis of the differences on fatigue and depression before and after between the two groups (*N* = 62).

Variable	Experimental group (*N* = 31)	Control group (*N* = 31)	*t*	*P*
	Posttest-pretest	Posttest-pretest		
	Mean (SD)	Mean (SD)		
Fatigue (TFRS)	39.71 (39.71)	70.35 (56.37)	−6.13	.001
Physical	15.90 (14.79)	26.00 (23.60)	−6.13	.001
Psychosocial	13.39 (19.06)	25.03 (20.29)	−6.87	.001
Daily activity	7.81 (10.97)	19.32 (16.21)	−6.64	.001
Depression (BDI)	6.73 (8.66)	5.34 (7.99)	−0.64	.525

TFRS: Tang Fatigue Rating Scale; BDI: Beck Depression Inventory.

**Table 4:** Changes in VAS fatigue and VAS depression within groups over 5 times (*N* = 62).

Variable		T1^a^	T2	T3	T4	T5	*F*	df	*P*
		M (SD)	M (SD)	M (SD)	M (SD)	M (SD)			
Fatigue^b^	Experimental group (*N* = 31)	3.12 (0.43)	3.28 (0.58)	3.58 (1.11)	3.91 (1.45)	5.25 (1.38)	16.907	4	.001
	Control group (*N* = 31)	2.58 (0.63)	3.72 (1.12)	4.12 (1.47)	4.73 (1.41)	6.15 (1.31)	48.559	4	.001

Depression^c^	Experimental group (*N* = 31)	3.06 (0.22)	3.06 (0.20)	3.09 (0.80)	2.98 (0.90)	3.67 (1.10)	4.904	4	.004
	Control group (*N* = 31)	3.11 (0.30)	3.45 (0.55)	3.54 (0.61)	3.95 (1.04)	4.32 (1.01)	14.374	4	.001

*Note*. ^a^Patients were followed up from initial treatment through the first five days of treating transcatheter arterial chemoembolization (TACE) (1, 2, 3, 4, and 5 days from treating TACE, respectively); T1 = 1 day from treating TACE; T2 = 2 days from treating TACE; T3 = 3 days from treating TACE; T4 = 4 days from treating TACE; T5 = 5 days from treating TACE; reference group was T1.

^b^Measured by VAS fatigue scale, range from 0 to 10.

^c^Measured by VAS depression scale, range from 0 to 10.
